# Perspectives on the popularization of smart senior care to meet the demands of older adults living alone in communities of Southwest China: A qualitative study

**DOI:** 10.3389/fpubh.2023.1094745

**Published:** 2023-02-24

**Authors:** Dehui Kong, Siqi Liu, Yan Hong, Kun Chen, Yu Luo

**Affiliations:** School of Nursing, Army Medical University (Third Military Medical University), Shapingba, Chongqing, China

**Keywords:** smart senior care, older adults, live alone, community, qualitative research

## Abstract

**Background:**

Older adults who live alone face challenges in daily life and in maintaining their health *status quo*. Currently, however, their growing demands cannot be satisfied with high quality; therefore, these demands expressed by elders may be settled in the form of smart senior care. Hence, the improvement in smart senior care may produce more positive meanings in promoting the health and sense of happiness among this elderly population. This study aimed to explore the perceptions of demands and satisfaction with regard to the provision of senior care services to the community-dwelling older adults who live alone in Southwest China, thus providing a reference for the popularization of smart senior care.

**Methods:**

This study adopted a qualitative descriptive approach on demands and the popularization of smart senior care. Semi-structured and in-depth individual interviews were conducted with 15 community-dwelling older adults who lived alone in Southwest China between March and May 2021. Thematic analysis was applied to analyze the data.

**Results:**

Through data analysis, three major themes and subcategories were generated: “necessities” (contradiction: more meticulous daily life care and higher psychological needs vs. the current lower satisfaction *status quo*; conflict: higher demands for medical and emergency care against less access at present), “feasibility” (objectively feasible: the popularization of smart devices and applications; subjectively feasible: interests in obtaining health information), and “existing obstacles” (insufficient publicity; technophobia; patterned living habits; and concerns).

**Conclusions:**

Smart senior care may resolve the contradiction that prevails between the shortage of medical resources and the increasing demands for eldercare. Despite several obstacles that stand in the way of the popularization of smart senior care, the necessities and feasibility lay the preliminary foundation for its development and popularization. Decision-makers, communities, developers, and providers should cooperate to make smart senior care more popular and available to seniors living alone, facilitating independence while realizing aging in place by promoting healthy aging.

## 1. Introduction

With the global aging process continuing to deepen, unprecedented attention has been given to older adults as well as to the demands for senior care. The process of population aging is faster in China than in most of the higher-income countries (1). Until 2020, the population of China aged 65 years and above accounted for 13.5% of the total population (2). This proportion is projected to increase to 26.1% by 2050 (3), which is marked as a super-aged society (4). With the expansion of life expectancy, life events, including changes in living arrangements, divorce and widowhood, and a large migration of the population to big cities, have increased the number of older adults living alone (5). In Western countries and some Asian countries such as China and Japan, the proportion of senior citizens living alone has increased (6). Compared with their counterparts living with families, those living alone are busy with several daily household chores (7) and are prone to develop chronic diseases (8). Research showed that older adults living alone were more likely to suffer from social isolation, to feel lonely, to develop depressive symptoms (9), and to receive less social support (10). Due to the lack of a companion, those elders who are living alone lack the encouragement to maintain a healthy lifestyle. In case of an emergency such as a sudden illness, they cannot have timely access to medical care (11), and the consequences may be fatal, resulting in higher medical costs and more meticulous care (12). In China, in the future, the number of senior citizens living alone will further increase, and more attention should be given to the vulnerable population (13). Being the primary place where the older adults live, the community plays a significant role in providing support. However, a survey in China noted that there was a low percentage of fulfilled needs, with services provided by communities being insufficient (14), among which regular visits by professionals, referrals, and first-aid, physical examination, and education were the most sought after needs in medical demand (15). Moreover, it has been indicated that, in the United States of America, Singapore, and China, senior citizens were more willing to take care of themselves at home or in the community, bringing a better quality of life, a sense of belonging, and security, thereby achieving social value (16–18).

Innovations and rapid advances in technology reformed modern medicine and health care for older adults (19). Smart senior care is a systematic, intelligent development of the conventional eldercare industry combining modern technology, aiming to enhance the living conditions and meet the needs of older adults in health management, safety and security, emergency aid, entertainment, and learning *via* technological services and products (20, 21). With the integration of information and technology such as big data, the Internet of Things, cloud computing, electronic health care, and mobile Internet, smart senior care is able to collect human signs and provide home care and interventions, realizing information interconnection among home, community, and medical institutions (22, 23). According to previous studies, smart senior care plays diverse roles in different scenarios with various devices. First, smartphone applications are used to obtain services. For instance, iFall and Smartfall are two Android apps for older adults which can send signals and alerts (24) as well as detect falls (25). iWander monitors the wandering elders with Alzheimer's diseases (26). More broadly, smart home technology is used for supporting older adults with dementia (27). Some smart home technologies can even control the environment such as giving injections of drugs such as insulin *via* actuators (28). Third, the application of artificial intelligence makes it possible for robots to attend to older adults with rehabilitation needs (29, 30). Fourth, the use of wearable sensors make remote monitoring possible, with functions of measuring vital signs in real-time, sending alerts in emergency circumstances (31), and intervening in a planned way in those circumstances (32).

As mobile devices and networks get popularized, the scope of application of smart senior care has become wider and wider, and the utilization rate has gradually increased. Studies revealed that older adults are usually positive toward gerontechnology, but they also raise questions and express their concerns on the ethics and technology adopted, such as autonomy, privacy and data protection, data accuracy and reliability, and other considerations such as biological effects and standardization of the devices and applications (33, 34). However, smart senior care still provides a new idea and option for senior care with the potential to promote safety and independence for the elders through discovering and preventing accidents, alerting, and locating (35). Through the monitoring of daily activities, abnormal behaviors such as falls, and cognitive anomalies, the wellbeing of the solo-living senior citizens can be guaranteed to a greater extent (36). Continuous monitoring is crucial for those living alone, which allows for the provision of the unremitting health status of older adults (37), realizing early detection and diagnosis of diseases, and can improve the quality of life and reduce the waste of medical resources (38). Moreover, the combination of interprofessional teams and smart senior care enables primary care physicians, nurses, specialists, and other professionals to discuss together to handle elderly patients with multimorbidity without transferring them (39), while avoiding multiple outpatient appointments as well as preventing the spread of inadequate and conflicting information between medical staff (40).

In response to the irreversible aging process, smart senior care may be an effective solution to promote healthy aging, which contributes to advancing the living standards and maintaining the functional ability and independence for community-dwelling older adults (22, 41). In spite of these advantages, to date, in China, the application of smart senior care is still at an early stage, with smart devices being relatively simple (42). Without satisfying and understanding the needs and expectations of older adults, smart senior care cannot be adopted undoubtedly (43). Thus, considering the demands and willingness of the elders to apply smart senior care is essential for its development (44). Accordingly, this study aimed to explore the perspectives of community-dwelling older adults who live alone with respect to demands and their satisfaction with regard to senior care by listening to the voice of the demanders so as to provide reference for the popularization of smart senior care.

## 2. Materials and methods

### 2.1. Study design

This article is a qualitative descriptive study which is widely used in the fields of medicine and health care and is also suitable for seeking information to develop suggestions or interventions (45). The study was facilitated through semi-structured one-to-one in-depth interviews.

### 2.2. Settings and participants

The study was conducted from March to May 2021. A purposive sampling method was employed to select the elders living alone in two communities of the main urban area in Chongqing as the research objects. Chongqing is a municipality located in the southwest part of China. As of the year 2020, the population aged 65 years and above accounted for 17.08% of the total population, which is higher than the average level of 13.5% in China (2). Before the interview, the research proposal was delivered to the staff of the community healthcare service centers to gain their collaboration to identify potential participants through their information system, and recruitment advertisements were simultaneously distributed in the communities. Older adults who were interested in participating in the study contacted the researchers directly or *via* the community staff. The inclusion criteria of subjects included seniors (1) aged 65 years or above; (2) who are living alone; and (3) who are able to understand the contents and express their thoughts. The exclusion criteria of subjects were as follows: (1) seniors with cognitive impairment and (2) those who were unable to cooperate.

### 2.3. Instruments

A semi-structured interview outline was designed through literature retrieval, group discussion, and pre-interview revisions. The sequence of questions raised was adjusted according to pre-interviews, and ambiguous expressions were modified, which mostly included: (1) How is your current living condition? Are there any difficulties regarding eldercare? What services do you need most presently? (2) How do you feel about living alone? In what ways do you usually communicate with others, and who are the most frequent persons you communicate with? How does the community help you with emotional needs? (3) Have you ever used/heard of any means of smart eldercare for registration, consultation, health-related queries, drug delivery, or making an appointment for health examination or healthcare services through the Internet? If yes, please provide more details. (4) If software or a function with positioning and alarm services in emergencies is accessible, are you willing to use it and if so, why? (5) Are you willing to apply smart senior care to your needs and what are its benefits/difficulties?

### 2.4. Data collection

Before formal data collection, the research purpose and contents were interpreted to the community-dwelling elders living alone. Meanwhile, we explained to the elders that the interview would be recorded and promised to keep this study confidential, strictly protecting the privacy of the subjects. Written informed consent was obtained thereafter. The demographic data were obtained before formal interviews were held, which were accomplished together with the same interviewer and recorder, DK and SL. The location of the interview was left to the discretion of the interviewees to choose a place that is quiet and familiar for older adults based on meeting the environmental requirements for interviews. The whole process was recorded synchronously using a voice recorder and non-verbal information, including expressions, body gestures, and tone, was observed and recorded in time. Various methods such as explanation, clarification, and questioning were applied during the interviews, and any inducement and suggestion were avoided. Each interview lasted for 20–40 min. When the interview was completed, the opinions of the participants were collected for a follow-up improvement. The sample size was considered until the data were repeated and no new topic emerged (46). After interviewing 13 older adults living alone, the data were saturated and no new content was included. We interviewed two more older adults living alone and confirmed that the data obtained were fully saturated. Finally, the data of 15 interviewees were analyzed.

### 2.5. Data analysis

Data collection and analysis were carried out simultaneously, and the recorded conversations were transcribed verbatim within 24 h of the interviews. Then, the transcripts were checked with the original recordings to ensure accuracy manually. Inductive thematic analysis was used to analyze the data manually, following the procedures proposed by Braun (47, 48): (1) Two researchers, DK and SL, read through the transcripts independently to get familiar with the contents. (2) They separately retrieved information related to smart senior care and the demands and satisfaction of the participants to form initial codes. (3) The two researchers classified the primary codes into potential themes separately and gathered related quotations. (4) In face-to-face meetings, the research team reviewed and verified the themes and split, merged, or deleted some topics according to the standards of internal and external heterogeneity to generate a thematic framework. Any disagreement on themes was discussed together to reach a consensus. (5) All of the researchers participated in clarifying the contents and developing a name for each theme. (6) The analysis report was written.

## 3. Results

In this study, 15 community-dwelling older adults living alone were interviewed and their average age was 75.07 years (range 68–79 years). Of the participants, seven were men and eight were women (Their details are presented in Table 1). Twelve out of the 15 participants suffered from chronic diseases, among which hypertension, diabetes, and cardiovascular diseases were the most common.

**Table 1:** Demographic characteristics of the participants.

**Variable**	**Number**
**Age**	
65–74	7
≥75	8
**Gender**	
Men	7
Women	8
**Pre-retirement occupation**	
Teacher	1
Worker	8
Civil servant	3
Engineer	1
Unemployed	2
**Education**	
Primary school	6
Junior high	4
Senior high	2
College and above	3
**Number of children**	
0	1
1	5
2	6
3	3
**Monthly income (CNY)**	
0–2999	2
3000–3999	10
≥4000	3
**Time living alone (year)**	
<1	2
1–5	8
≥6	5

**Figure 1:**
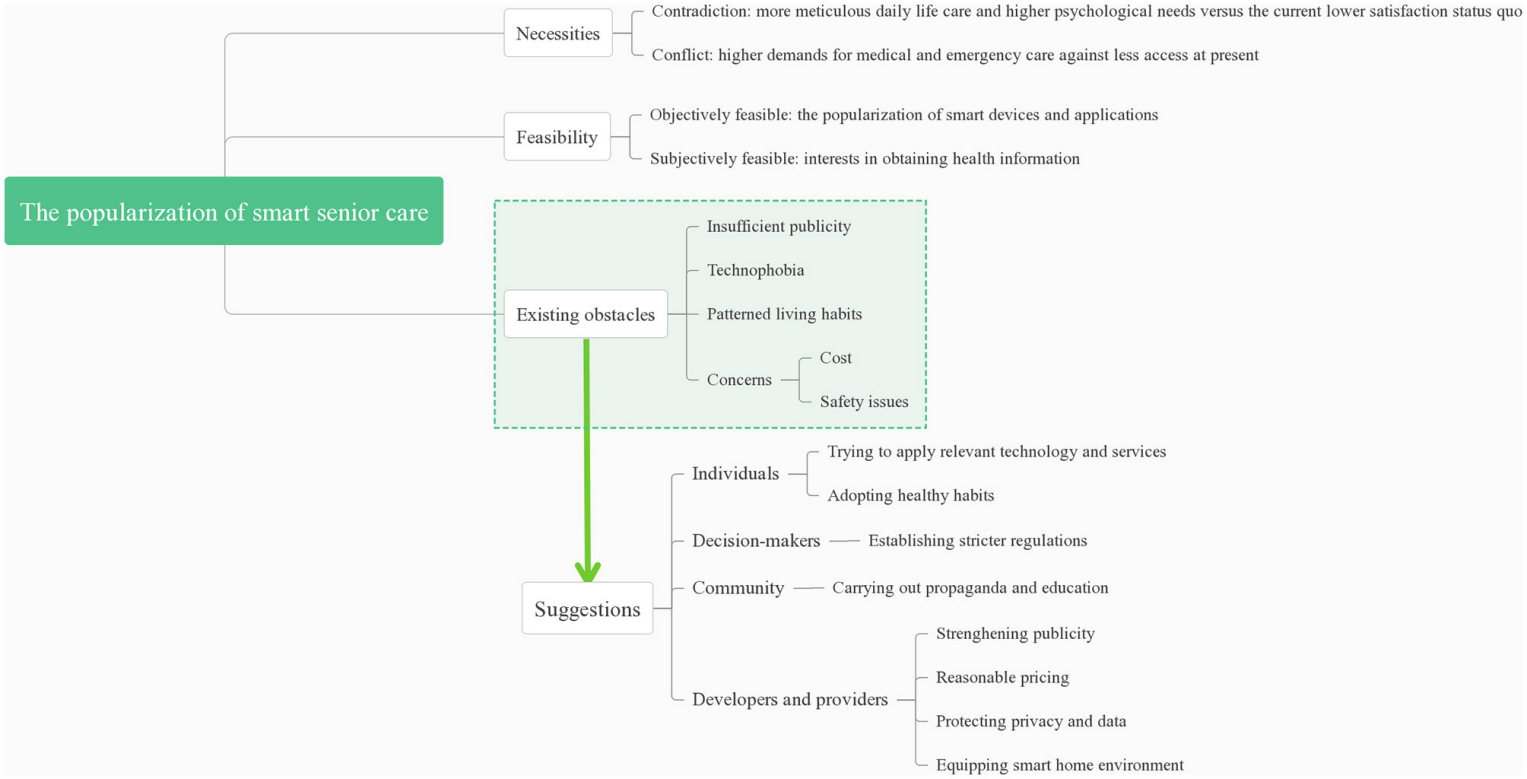
Domains of the themes and suggestions on the popularization of smart senior care.

Based on the perspectives to meet the demands of senior citizens living alone in communities in Southwest China, the popularization of smart senior care could be summarized into three themes: necessities, feasibility, and existing obstacles. The domains of the themes are demonstrated in Figure 1.

### 3.1. Necessities

The participants expressed their needs in daily life and psychological aspects as well as in medical and emergency care, but they hardly received any related services.

#### 3.1.1. Contradiction: More meticulous daily life care and higher psychological needs vs. the current lower satisfaction status quo

The elders living alone reported a high demand for daily care services, but few services were provided to them presently, and the difficulties faced in rendering elderly care were concentrated. *P12: “It would be better if there was a canteen for us seniors. We could eat there and then go back home to rest.” P13: “My wife was in hospital for 10 days due to cardiovascular diseases and spent more than 100,000 yuan in a short time last year. When older adults are ill, the biggest difficulty is the financial problem.” P14: “I'm afraid of inability to take care of myself, completely bedridden one day. By then what can I do?”*

The elders living alone were reluctant to place the full burden of care on their children who were also shouldering the burden of both work and family. They required supplemental care to compensate for home care. *P13: “Seniors at our age often have only one child. The only child of the family lives separately who also must raise children, and they cannot spare more time and energy to take care of the older generation. So, there are more older adults who need help and care. Once sick, it is hard to know if one is alive, what the physical condition is. It is even difficult to seek help.”*

Loneliness was common, and social interaction with peers was prominent. Among the interviewees, seven complained of being “lonely” and three mentioned “being used to it.” *P3: “The circle is smaller, and friends are fewer. Basically, there is no one with the same hobbies. People from the same work unit and at the same generation have long gone if older than me, and I don't know the ones younger than me because I retired earlier.” P13: “After my wife left, I was not used to it for a long time. She had lived with me for decades, and it's too lonely to live alone. There is no companion around. I'm the only one eating at the dining table, only one bowl and one pair of chopsticks from morning till night. I've been living alone for almost a year. It's getting better now.”*

Social interactions with peers, such as former colleagues, neighbors, and friends, were of great significance to senior citizens living alone. *P1: “As long as the neighbors don't see me in one day, they will call and ask me where I am. We, several teachers often get together and play, cards for example.”*

#### 3.1.2. Conflict: Higher demands for medical and emergency care against less access at present

In this study, the elders living alone expressed strong demands for medical and emergency care, whereas present services are limited and insufficient. *P11: “Recently, the community staff came but only had a look. They took at most a simple device like blood pressure meter which I had already bought.”* Some participants also questioned the medical skills of community physicians, and they thought whether to accept the services or not depended on disease conditions. *P13: “It is likely to visit the community physician for some common illnesses, but not for severe diseases.”*

All the participants considered the software that could realize a one-click alarm in the case of emergency as necessary, which could save lives at a critical moment, and expressed their willingness to use it in future. *P10:* “*It is necessary. Since I live alone, if I get sick at night suddenly, it would be a big trouble. I am on the 7th floor, by then I can't go downstairs. So, I need this.”* As for medical alarm systems such as the fall or immobility alarm, most seniors said it was useful “just in case.” *P2: “This is still necessary. It is useful in emergency and just in case.” P8: “It should be used. Gao jiahu was in our group of the factory. After his death, all the maggots crawled out, and no one was at home. So, after my wife passed away, neighbors asked me to come down to show up every day, and they could know how I was.”*

### 3.2. Feasibility

Smartphones and some applications were prevalent among older adults in this interview. The participants were not only willing to receive health-related information but were also willing to take up the initiative to inquire.

#### 3.2.1. Objectively feasible: The popularization of smart devices and applications

Ten of the seniors living alone in this interview used smartphones, which were mostly purchased by their children. Additionally, all those who had smartphones could use applications such as WeChat for daily chats and video functions. Several of them could also use social interaction and health management functions. *P1: “I can use WeChat video and voice calls. When I go out to play with friends, I will post all photos on Moments. My previous students will definitely “like” them when they see the pictures I post.” P10: “I can video chat and post photos on Moments, and I also use WeChat sports every day. For example, I will know how many steps my son and granddaughter walk in a day, whether they are busy or not. Sometimes when they walk too much, I even joke and ask where you have gone.”*

#### 3.2.2. Subjectively feasible: Interests in obtaining health information

The elders could obtain health-related information through various channels, and the majority of them expressed their willingness to receive and actively inquire about health information, especially the contents related to their health and diseases, but they had difficulty in discriminating the quality and accuracy of related information. *P4: “I am eager to know what the professors talk about, and the information related to high blood pressure.” P8: “I usually search for health information with Baidu, but it should be read selectively, as some are advertising. I read books mostly because I have noticed irresponsible information posted on Baidu. A book is more responsible, since editors have reviewed and audited it after all.” P15: “I read health information. I have to evaluate if it is useful and real, or for selling medicine. There are many advertisements. I doubt if it is real.”*

### 3.3. Existing obstacles

Although the elders living alone expressed high demands yet low satisfaction, smart senior care has not been regarded as a settlement to meet their needs. We also explored the probable reasons for this contradiction.

#### 3.3.1. Insufficient publicity

The interviewees were not aware of most services of smart senior care. When asked about online consultation, health-related queries, having an appointment for health examination, and booking healthcare services through smartphones or the Internet, most of the participants had never used or heard of such services. In terms of online registration, most of the participants had merely heard of the related information from offspring who had registered online for them. *P8: “I don't know how to register online to see a doctor. But not long before my granddaughter registered online for me when I went to Xinqiao hospital.”* As regards online drug delivery, only one participant had ordered medicines online by herself, while the son of another participant had ordered medicines for her through the Internet. *P11: “I often get medicines online. My son buys them for me.”*

#### 3.3.2. Technophobia

Most of the seniors included in the present study were motivated to learn the functions of a smartphone they needed and were interested in, but still they were able to use only a limited number of applications. *P7: “I use a smartphone for investing in stocks. I'm willing to learn useful functions if I like it, but I need someone to teach me.”*

However, most of the participants were intimidated by the relatively complex functions of smartphones and applications. They refused to use a smartphone for fear of being cheated and thought that it was cumbersome and complicated. *P2: “I don't use WeChat since seniors are afraid of being fooled. Simple functions are OK. I know it's very troublesome to chat on a cell phone through WeChat, TicTok, etc. I don't even think about it or browse it.” P15: “I use a cell phone for seniors. My child suggested replacing a smartphone for me, but there are too many new functions and I'm not able to use it. I am too lazy to learn.”*

#### 3.3.3. Patterned living habits

Older adults living alone had some unhealthy living habits. Even if they were aware that these habits were harmful to their health, making changes to their diet pattern was still difficult for them, such as excessive salt intake and eating leftovers. *P13: “Salt intake usually exceeds the limit, and 5 g salt a day is just too little. To prepare only two or three dishes in one meal will exceed the limit. When less salt is added to the dishes, it is not tasty, with no appetite at all. The physicians also told us that salt consumption couldn't exceed 5g, but no one can do it.” P14: “I know it's not healthy reheating leftovers, but there are usually too many, and it's wasteful to dump the dishes.”*

#### 3.3.4. Concerns

Cost was the topmost consideration as regards whether to accept related services or not. *P11: “It is useful, but cost money. We must save money for hospitalization and surgery. For example, when one is hospitalized and told he has cancer, he must spend money at that time. So, I should be more careful at this point.”*

Another consideration concentrated on safety issues. First, as regards online payment, senior citizens living alone were less agreeable to make payments online. *P12: “I can use WeChat payment, but I don't use it frequently. I don't think it's safe enough and it might have kinds of hidden trouble.” P13: “I use cash. I'm afraid of making mistakes, because I'm at an old age, and sometimes I may make a mistake on a decimal point. I can't even figure out what's wrong with the payment.”*

Second, referring to online drug delivery, which is better known, the elders had a skeptical or negative attitude, worrying about the security aspect. *P1: “I have never bought drugs online. I don't believe it. I think it is better to prescribe medicine in a hospital. I am afraid to buy fake medicine.” P11: “I am worried to be cheated, fearing that the medicine is advertised to be good but fail to achieve the desired effect. I like it (buying medicine online). But I am afraid of being fooled. If the medicine is to be sold online, it should be reviewed repeatedly and gain approval and we will be at ease when taking medicine.”*

## 4. Discussion

The contradiction between the shortage of experienced staff and the growing demands for eldercare is becoming increasingly prominent. It is difficult to fully rely on manpower to provide care services for older adults (28, 44). Smart eldercare services may assist community-dwelling older adults who live alone in many ways. Physically, the fulfillment of their willingness to age in place requires support in daily life (49). This is consistent with other studies at home and abroad that receiving daily life care helps to improve the quality of life, thus increasing satisfaction (50–52). However, participants in this interview are unwilling to put all care burden on their offspring since they do not want to seem burdensome, similar to the surveys referring to the refusal to be troublesome and loss of control (53, 54) but promoting the use of technology (55). Consequently, smart senior care is of vital importance as an alternative or supplement to family care for solo-living older adults. Psychologically, despite the elders living alone often feeling lonely, there is a lack of community attention to their mental health, which is often overshadowed by physical health (56). Our interview coincides with previous studies that online social interaction with peers plays an essential role in the interpersonal relationships of older adults living alone, increasing their social involvement and enhancing their sense of happiness (57, 58).

In addition, older adults living alone require continuous and immediate medical support, and some dangerous conditions leading to deterioration in their health can be avoided through real-time monitoring (28, 59). The need for medical care of senior citizens living alone is higher, whereas corresponding services provided by the community are basic and limited. It was reported that Chinese older adults needed health monitoring, drug delivery, and home visits (60). Additionally, the emergency care demands of the elders living alone are particularly significant. Unlike some other countries (36, 61), present-day China lacks long-term telecare such as remote consultation and one-click alarm calls for help (62). With the development of science and technology, smart senior care is expected to provide medical services and emergency rescue measures, filling the gap in family care, thus creating a safe and secure environment for older adults who live alone (63).

The feasibility of the popularization of smart senior care consists of two aspects. First, with the spread of the Internet, an increasing number of senior citizens were involved in the wave of science and technology in China (13). The Statistical Report on China's Internet Development pointed out that the percentage of middle-aged and elderly netizens with Internet access in China has grown at the fastest pace. As of June 2021, the number of Internet users aged 60 years and above exceeded 100 million, accounting for 12.2% of the total Internet users, with an increase in 1.9% compared with the same period of 2020 (64). Moreover, the COVID-19 pandemic motivated senior citizens to use smart devices, similar to their Chinese counterparts, senior citizens in the USA used computers more frequently than prior to the epidemic, owing to various reasons such as more reliance on telemedicine (65). Furthermore, a growing number of health management applications are available online as well (66). Second, our study reveals that older adults who live alone are actively interested in receiving and obtaining health-related information, in line with an Australian study which found that most of the older adults took interest in accessing websites on healthy aging (67). A study also showed that nearly half of the Chinese seniors surfed online daily and showed more interest in health-related information, with Internet access becoming the most likely way to fetch health-related information (59). In the process, senior citizens also had questions on information quality, which put forward higher requirements for the authority and reliability of health information on the Internet. The initiative of the elders to pose health-related queries has become another motivation for the popularization of smart senior care (68), which makes it possible to enter into the community and the life of senior citizens living alone.

However, there are still barriers against the popularization of smart senior care. The first affecting factor is related to insufficient publicity, bringing about the incuriosity of older adults in the first place. Except for drug delivery and online registration, the interviewees never heard of services related to smart senior care, not to mention practical utilization. Low awareness will inevitably bring low usage. In addition, as Smith B et al. pointed out, although more access to health information is available now than ever before, large amounts of information without adequate explanation and guidance could lead to confusion (69). Therefore, a proper advertising with clear instructions is prerequisite. The second affecting factor pertains to the fact that, as a technologically “marginalized” group, older adults are still relatively unfamiliar with smartphone applications (61). Similarly, an American survey reported that older adults needed more time to learn how to use such technologies than the younger (70). Even the elders with relevant experiences still had difficulty in carrying out basic operations (71). Due to their negative attitudes toward intelligent products and functions as well as the limited information received, older adults believe that they are incapable of handling technological products, resulting in “technophobia” and unwillingness to learn the applications, thereby generating a vicious circle (72). The third affecting factor mentions that, although older adults have multiple accesses to health information, such information does not fully convert into healthy behaviors. Even being aware of the fact that certain behaviors are unhealthy, it is hard for older adults to alter them sometimes because these habits formed over a long term and there was no assistance from cohabitants. A Canadian survey with more than 75% of the participants living alone also revealed that unhealthy eating habits were associated with low health literacy and economic status (73). Consequently, when it comes to the solo-living seniors, smart senior care is likely to take an active part in sending continuous reminders and instructions to them. The fourth affecting factor is related to concerns. The primary concern is cost, which is rooted in the traditional concept of “economy” in China. If the service is not cost-effective, the elders would rather spend money on items that are more necessary in accordance with a survey in the UK (66). Another concern is associated with security issues, which were also reported in a previous study (63, 70). Considering limited guidance to help the elders, coupled with their lack of knowledge about details in smart eldercare services (74), it is rational to worry about “being cheated.”

To remove the obstacles, we can formulate propositions in four aspects. As for decision-makers, stricter regulations for smart senior care services can help alleviate the worries of senior users. Additionally, if smart senior care could be covered by health insurance, older adults are more likely to adopt such services (34), especially for the solo-living. From the communities' side, propaganda and education ought to be carried out routinely to advance the digital health literacy of older adults who live alone with no one nearby to teach them. Developers and providers have to consider the fact that, only by strengthening the publicity of smart senior care can the awareness of the solo-living elders be increased. Figuring out a rational way to advertise is an indispensable option. In addition, the price of the services should be affordable for senior citizens, with the security aspect taken into consideration. Additionally, when it comes to ethical issues, more attention should be paid to privacy and data protection to ensure the autonomy and dignity of older adults, such as restraining information sharing, introducing data ownership policies, and facilitating control over technology by older adults themselves (34, 75). Moreover, home environment with alarm systems, sensors and actuators, activity recognition, monitoring and communicating systems, and Internet connection should be taken into consideration during the construction of buildings and renovation of houses (76). For the elders living alone, adopting healthy habits, discarding their prejudice, and attempting to apply relevant technology and services are likely to be the first steps.

## 5. Conclusion

This study investigated the senior care needs of community-dwelling older adults living alone and their perspectives on smart senior care. At present, the *status quo* of unmet needs for daily life care, psychological, medical, and especially emergency care motivates the application of smart senior care in this solo-living group. In recent years, the popularity of smart devices and software has made it possible for older adults to access technology and related services. In addition, their initiative to obtain and query health-related information lays the foundation for the popularization of smart senior care. However, older adults still have some concerns over smart senior care, which are becoming the existing obstacles to its promotion. In this regard, joint efforts should be made to address the barriers. Older adults who live alone ought to break down the stereotypes of technology and adopt healthy habits. Decision-makers, communities, developers, and providers should coordinate to make smart senior care more well known and accessible, offer related services to older adults, particularly to those living alone, realizing aging in place, thereby responding to international calls for healthy aging. The results of this study will also provide reference for the popularization and development of smart senior care.

### 5.1. Strengths and limitations

The number of older adults living alone has witnessed a rapid surge in China (77). This study highlighted the elders living alone, as few previous research studies reported, to call on public attention to this population. Moreover, limited qualitative research has been conducted on demands and the popularization of smart senior care. Conversations can get closer to the real feelings and the inner voice of older adults. We also hope that, in this way, the elders can participate in the planning of eldercare as co-designers instead of being passive recipients.

This study also has a few limitations. First, the sample size of this study was limited and was mainly taken from the central urban areas of Chongqing. The results may not be generalized to the national level. Furthermore, given cultural differences, the reasons for older adults living alone in China may be different from those living in Western countries. In China and other Asian countries, living with children is still the first choice for most of the older adults, while living alone is often the last resort for them. Instead, most senior citizens in Western countries believe that living alone gives them more independence and can protect their personal privacy (78), which is more of an individual's active choice.

## Data availability statement

The raw data supporting the conclusions of this article will be made available by the authors, without undue reservation.

## Ethics statement

The studies involving human participants were reviewed and approved by the Ethics Institutional Review Board of Army Military Medical University/Third Military Medical University (approval number 2021-13-01). The patients/participants provided their written informed consent to participate in this study.

## Author contributions

DK: study design, data collection, interpretation and analysis, and manuscript drafting. SL: data collection, interpretation and analysis, and manuscript drafting and revision. YH: study coordination, data analysis, and manuscript revision. KC: study design, data analysis, and manuscript revision. YL: guarantor of integrity of the entire study, study design, and manuscript revision. All authors read and approved the final version of the manuscript.
